# Exploring the Core Formose Cycle: Catalysis and Competition

**DOI:** 10.3390/life14080933

**Published:** 2024-07-25

**Authors:** Jeremy Kua, L. Philip Tripoli

**Affiliations:** Department of Chemistry and Biochemistry, University of San Diego, San Diego, CA 92110, USA

**Keywords:** origin of life, thermodynamics, kinetics, prebiotic chemistry, formose reaction, sugars

## Abstract

The core autocatalytic cycle of the formose reaction may be enhanced or eroded by the presence of simple molecules at life’s origin. Utilizing quantum chemistry, we calculate the thermodynamics and kinetics of reactions both within the core cycle and those that deplete the reactants and intermediates, such as the Cannizzaro reaction. We find that via disproportionation of aldehydes into carboxylic acids and alcohols, the Cannizzaro reaction furnishes simple catalysts for a variety of reactions. We also find that ammonia can catalyze both in-cycle and Cannizzaro reactions while hydrogen sulfide does not; both, however, play a role in sequestering reactants and intermediates in the web of potential reactions.

## 1. Introduction

It is curious that autocatalytic cycles are ubiquitous in living systems [1] but rare in benchtop chemistry. The oligomerization of formaldehyde (CH_2_O) into sugars via aldol reactions, known as the formose reaction, is one of the rare exceptions. Its mechanism was first elucidated in 1959 by Breslow [2], with an update in 2014 [3], and has been extensively studied for its potential in food production [4]. While often mentioned in origin-of-life chemistry as a “simple” route to ribose (and other sugars utilized in extant biochemistry), the formose reaction typically forms a complex and intractable mixture under (catalytic) alkaline conditions [5] with acids and alcohols produced via the competing Cannizzaro reaction.

Early prebiotic chemical syntheses focused on finding conditions to increase ribose selectivity; three examples are using phosphates [6], borate [7], or trying to find the best metal catalysts [8]. But more recently, the messiness of the formose reaction is embraced as a feature, not a bug. The Huck group systematically probed how environmental conditions affect the observed product distribution [9,10,11]. Paschek et al. investigated the effect of catalysts that might be present in carbonaceous chondrites [12], Omran considered chemical gardens resembling hydrothermal vents [13], Vinogradoff et al. utilized olivine silicate catalysts [14], and Haas et al. examined mechanochemical effects [15].

The formose reaction also caught the attention of computational modelers as a classic test case to examine chemical reaction networks. Examples include heuristics-aided quantum chemistry [16], graph-theoretical approaches coupled with quantum calculations [17], and utilizing machine learning on reaction-rule networks [18]. These large-scale studies took a bird’s-eye view, showcasing the complexity of the system with its myriad compounds, but do not focus on individual reactions or how molecules in the network may act recursively as catalysts. Our present work complements these approaches by examining in detail one small portion of the formose reaction—its smallest autocatalytic core. At this small yet focused scale, we previously generated a free energy map (thermodynamics and kinetics) of the uncatalyzed formose reaction [19]; our present work extends the earlier work by examining the effect of catalysts and exploring reactions that may sequester reactants and intermediates in the smallest autocatalytic cycle.

**Figure 1:**
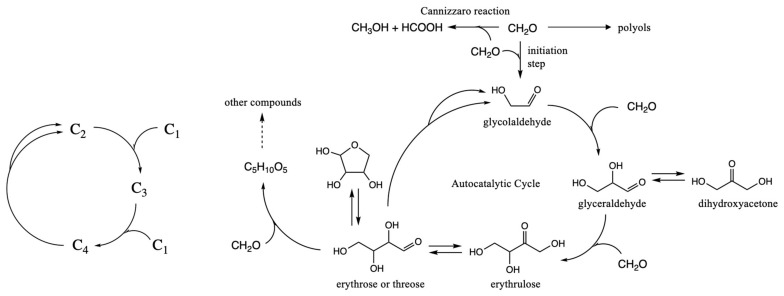
Core autocatalytic cycle of the formose reaction.

The smallest autocatalytic cycle of the formose reaction is shown in Figure 1. CH_2_O is the C_1_ “food” species. The linchpin C_2_ species is glycolaldehyde. C_3_ and C_4_ species can be produced via successive aldol addition of C_1_ food; larger sugars are also synthesized via this route. The key to autocatalysis is that C_4_ can undergo a retro-aldol reaction to produce two linchpin C_2_ species, thereby accelerating the consumption of C_1_ food with each successive turn of the cycle. In the formose reaction, myriad aldol condensations and retro-aldol reactions take place; for example, you could have C_2_ + C_3_ → C_5_ or C_6_ → C_3_ + C_3_, although a core autocatalytic cycle can be achieved with just C_1_ through C_4_ species.

Several features of this core cycle deserve mention, and we will make reference to our earlier work [19] on the free energy map of the thermodynamics and (uncatalyzed) kinetics of these reactions. Forming the C_2_ linchpin directly from CH_2_O (the initiation step) is very challenging kinetically because, without an umpolung species, it is difficult to make C–C bonds; our previously calculated barrier is 45.3 kcal/mol. However, once a small amount of the C_2_ linchpin is formed, the direct dimerization is bypassed. Food is consumed via the much lower barrier C_2_ + C_1_ → C_3_ and C_3_ + C_1_ → C_4_ reactions. The retro-aldol C_4_ → C_2_ + C_2_ reaction regenerates (more) C_2_ and accelerates the consumption of C_1_. As the food supply dwindles, a wider variety of sugars in the C_5_ to C_7_ range are produced.

Off-cycle reactions can deplete the concentrations of the C_1_ to C_4_ species involved in the cycle. The Cannizzaro disproportionation reaction parasitizes the cycle by removing CH_2_O food, but by producing HCOOH, it may add a catalyst to enhance reaction rates within the cycle. (The C_2_, C_3_, and C_4_ sugars can also undergo Cannizzaro reactions.) All the aldehydes can form polyols by oligomerization reactions forming C–O bonds. This could sequester some of the reacting species, slowing down reactions in the cycle, but also reduce parasitic reactions and provide an equilibrating pool of reactants, thus lengthening the lifetime of autocatalytic cycles. Keto-enol isomerization reactions (e.g., glyceraldehyde to dihydroxyacetone for the C_3_ species) or ring closures (e.g., erythrose or threose for the C_4_ species) may play similar sequestering roles.

Our previous free energy map only considered uncatalyzed reactions in aqueous solution under neutral conditions. The present work considers whether other small molecules may act as catalysts to influence the kinetics or act as sequestering agents to influence the thermodynamics of the reactions involved in (or adjacent to) the core cycle. The small molecules being considered in this article are (1) the C_1_ products of the Cannizzaro reaction CH_3_OH and HCOOH; (2) small molecular acids that may be found in hydrothermal vent reactions, such as carbonic acid and acetic acid; and (3) small molecules that provide reduced nitrogen and sulfur, NH_3_ and H_2_S, that may have been present at life’s origin.

To keep this investigation tractable, there is much we excluded. The rich chemistry of HCN (and formamide) is the subject of a future paper. Cyanide provides an umpolung C_1_ reactant and combines readily with aldehydes, making C–C bonds, and the plethora of intermediates increases many-fold. HCN is also a “high energy” reactant compared to the carbon and nitrogen sources that are prebiotically plausible: CO_2_ and NH_3_. Our calculated free energy change for the reaction CO_2_ + NH_3_ + H_2_ → HCN + 2 H_2_O is +18 kcal/mol. (Carbon monoxide is also excluded in this study and may be considered a cyanide analog). We have also excluded ions because the results are less than satisfactory with our current computational protocol (see Section 2 below). Finally, we limit ourselves to just the C_2_ to C_4_ “sugars” in the smallest autocatalytic cycle, as shown in Figure 1.

This article is organized as follows: After describing our computational protocol and its limitations, the combined Results and Discussion section (Section 3) will go through each of the reactions relevant to Figure 1, starting with C_1_ and working our way through the C_2_, C_3,_ and C_4_ reactions. Finally, we will consider sequestering the sugars by direct reaction with NH_3_ and H_2_S.

## 2. Computational Methods

Since we will be comparing our present calculations to our previous work, we apply the same computational protocol [19]. Here, we provide a brief description of that protocol for the convenience of our readers. Some of the text in this section is reproduced from our recent work on CHO systems published in this journal [20] since we think that description is both clear and succinct. Essentially, we calculate the free energies in an aqueous solution using quantum chemical methods, and our protocol shows suitable agreement with available experimental results for CHO systems [19,20,21,22]. Here are the computational details:

The geometry of each molecule is optimized, and its electronic energy is calculated at the B3LYP [23,24,25,26] in favor of density functional theory with the 6-311G** basis set. To maximize the probability of finding global minima, multiple conformers are generated using molecular mechanics (MMFFs force field [27]). The optimized structures are embedded in a Poisson–Boltzmann continuum to calculate the aqueous solvation contribution to the free energy. While this does not provide a specific concentration, it assumes a dilute solution such that the electrostatic field generated by a neighboring solute molecule is effectively screened by the water solvent. One can consider all solutes to have the same relative concentrations in our calculations. Zero-point energy corrections are included, and we apply the standard temperature-dependent enthalpy correction term (for 298.15 K) from statistical mechanics by assuming translational and rotational corrections are a constant times *kT* and that low-frequency vibrational modes generally cancel out when calculating enthalpy differences. So far, this is standard fare.

However, entropic corrections in aqueous solution are problematic [28,29,30]. Changes in free energy terms for translation and rotation are poorly defined in solution due to restricted complex motion, particularly as the size of the molecule increases (thus increasing its conformational entropy). Free energy corrections come from two different sources: thermal corrections and implicit solvent. Neither of these parameters is easily separable, nor do they constitute all the required parts of the free energy. We follow the approach of Deubel and Lau [31], assigning the solvation entropy of each species as *half* its gas-phase entropy (calculated using standard statistical mechanics approximations similar to the enthalpy calculations described above), based on proposals by Wertz [32] and Abraham [33] that upon dissolving in water, molecules lose a constant fraction (~0.5) of their entropy.

To estimate activation energies, transition states were optimized by including several explicit water and/or catalytic molecules to aid in transferring H moieties. All calculated transition states have one significant negative eigenvalue corresponding to the reaction coordinate (eigenvector) involving bond breaking/forming. Several conformers are tested in each case, and we only report the lowest calculated barriers.

When put to the test by first calculating the equilibrium concentrations in a self-oligomerizing solution of 1 M glycolaldehyde at 298 K, our protocol fared very well compared to subsequent NMR measurements [22]. Our relative Gibbs free energies in aqueous solution are typically within 0.5 kcal/mol compared to experimental results. That being said, our protocol did show systematic errors of 2–3 kcal/mol when calculating barriers involving carbonyl chemistry when compared to experimental results. Going to a higher level of theory does not reduce this error [34]. For the few S_N_2 reactions we considered, having to daisy chain an additional 3–4 water molecules to facilitate proton transfer results in barriers that may be up to 10 kcal/mol too high, consistent with our previous work [19,35]. Using carboxylate anions with diffuse functions to represent weak acids gives poorer results compared to keeping the COOH group neutral [20]. Our previous attempts to incorporate H_3_O^+^ (and its larger cousins) as catalysts also gave unrealistic barriers and structures, with transition states quickly falling into local minima [36]. We have also attempted using Group I or Group II metal cations in conjunction with hydroxide as catalysts (unpublished results) that run into similar issues.

Quantum chemistry is about error cancellation, and our protocol (with its foibles, including the simplistic entropy correction) has worked well even with this systematic error for activation barriers. Thus, we do well on thermodynamics and just okay on kinetics (but at least we are in the ballpark for the majority of the carbonyl chemistry and aldol reactions in this work), and we want to be very clear about both the opportunities and the limitations of our protocol in this brief description.

## 3. Results and Discussion

Throughout this paper, for the thermodynamics of a chemical reaction, the reaction free energy will be designated Δ*G*. For a transition state, kinetic barriers will be designated Δ*G*^‡^ and will typically refer to the forward direction. From the Δ*G* and Δ*G*^‡^ values for the forward reaction, the thermodynamics and kinetics of the reverse reaction can be easily ascertained. All numerical values are in kcal/mol, abbreviated as kcal. In each subsection, we begin with the uncatalyzed reaction (water may facilitate proton transfer) before examining the inclusion of a potential catalyst. The potential catalysts in this study are NH_3_, H_2_S, methanediol (the hydrated form of CH_2_O, which is dominant in aqueous solution), the C_1_ Cannizzaro products CH_3_OH and HCOOH, along with three other acids (carbonic, acetic, and glycolic) that are likely to be present in prebiotic systems.

### 3.1. Formaldehyde Hydration

As the main “food” species, CH_2_O in aqueous solution is typically hydrated and exists primarily as methanediol, CH_2_(OH)_2_. The reaction is as follows:

CH_2_O + H_2_O → CH_2_(OH)_2_ Δ*G* = −4.6 kcal

**Figure 2:**
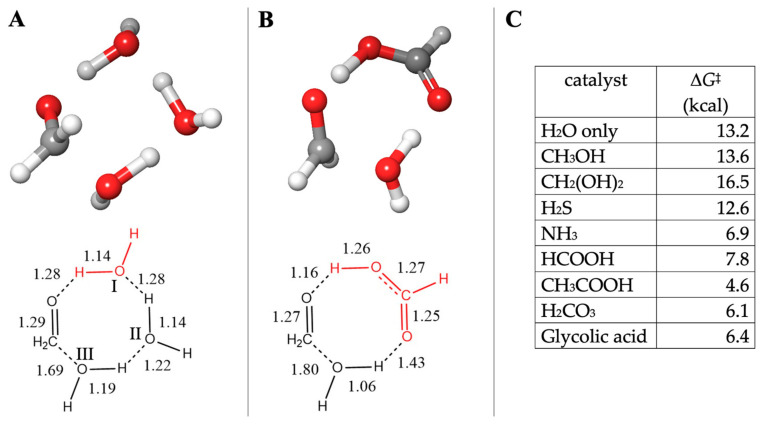
Transition state structures (**A**,**B**) and barriers (**C**) for formaldehyde hydration.

The optimal uncatalyzed transition state includes two *additional* explicit water molecules forming an eight-center transition state, as shown in Figure 2A; the barrier Δ*G*^‡^ is 13.2 kcal. The reactant water molecule is labeled III, and the additional water molecules are labeled I and II. Both our Δ*G* and Δ*G*^‡^ values are in good agreement with experimental results, as discussed in our previous work [19].

To investigate if prebiotically plausible small molecules potentially present in the solution can catalyze this reaction, we replace the water molecule I with CH_3_OH, CH_2_(OH)_2_, NH_3,_ or H_2_S. (Replacing the water molecule II is either less or equally favorable.) Or we can replace both water molecules I and II with a carboxylic acid moiety, as shown in Figure 2B with formic acid. This optimal transition state structure is geometrically similar to the eight-center water-only transition state in Figure 2A, but there are some notable differences in the bond distances. The acidic proton of HCOOH is 1.16 Å to the carbonyl-O, while the corresponding distance is 1.28 Å for the uncatalyzed reaction. The hydrating water molecule has a longer C…O distance of 1.80 Å instead of 1.69 Å in the water-only transition state. These are consistent with an acid-catalyzed mechanism where the proton transfer proceeds ahead of the nucleophilic addition of the reactant water to CH_2_O.

From the table in Figure 2C, we see that CH_3_OH, CH_2_(OH)_2,_ and H_2_S have no catalytic efficacy for formaldehyde hydration. The barriers are either similar to or greater than the uncatalyzed Δ*G*^‡^ of 13.2 kcal. As a proton transfer agent, NH_3_ substantially reduces the barrier. For the four carboxylic acids tested, the barrier is approximately halved. This fits well with NMR studies on the hydration and dehydration of formaldehyde [37], where the observed exchange rate constants (*k*_ex_ = *k*_hyd_ + *k*_dehyd_) were ~16 s^−1^ at pH 7.4 and range from ~4 s^−1^ in the pH 3–6 range (barriers are not provided in that work, but since ln 16 = 2.77 and ln 4 = 1.39, one might surmise that the barrier is approximately half in the acidic pH range). The order of our calculated Δ*G*^‡^ for the acids is likely not significant, given the computational error, and our later results will see some swaps in the order. Thus, we cannot relate the *p*K_a_ or an acid to its catalytic ability in this reaction. (The NMR results also do not show much distinction in the pH 3–6 range.)

Our results are consistent with experimental observations that under neutral or acidic conditions, in addition to CH_2_(OH)_2_, formaldehyde can oligomerize via C–O bond formation to form acetals, hemiacetals, and oxane rings [4] at higher concentrations of CH_2_O. This begins with the dimerization of formaldehyde to form the hemiacetal.

CH_2_O + CH_2_(OH)_2_ → HOCH_2_OCH_2_OH Δ*G* = −4.0 kcal

But since this is slightly less exergonic than formaldehyde hydration, at least under the “dilute” conditions of our computational protocol, the equilibrium will favor monomers, and the dimerization of CH_2_(OH)_2_ would be marginally endergonic.

2 CH_2_(OH)_2_ → HOCH_2_OCH_2_OH + H_2_O Δ*G* = +1.2 kcal

The formation of the six-membered trioxane (CH_2_O)_3_ and larger oxane rings is also endergonic relative to monomeric aqueous CH_2_(OH)_2_, as shown in our previous work [19].

In commercial use, methanol is typically added to aqueous formaldehyde solutions to prevent higher oligomers from forming. From our calculations,

CH_2_(OH)_2_ + CH_3_OH → CH_3_OCH_2_OH + H_2_O Δ*G* = −1.4 kcal

CH_3_OCH_2_OH + CH_3_OH → CH_3_OCH_2_OCH_3_ + H_2_O Δ*G* = −1.4 kcal

and thus, methanol acts as a capping group by favorably forming acetals. This suggests that the effective concentration of formaldehyde in aqueous solution can be lowered when a sequestering agent such as CH_3_OH is present. Since CH_3_OH is produced in the Cannizzaro reaction (see next subsection), we expect it to be present in prebiotic mixtures containing CH_2_O. We will consider the sequestering of the aldehydes in more detail later in this article.

### 3.2. The C_1_ Cannizzaro Reaction

Formaldehyde can disproportionate into methanol and formic acid. The reaction is thermodynamically favorable and catalyzed under alkaline conditions [5].

CH_2_O + CH_2_(OH)_2_ → HCOOH + CH_3_OH Δ*G* = −19.6 kcal

**Figure 3:**
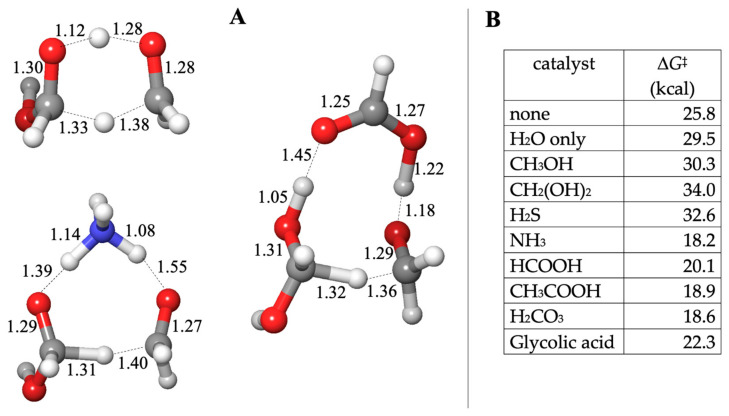
Transition state structures (**A**) and barriers (**B**) for the C_1_ Cannizzaro reaction.

The lowest energy transition state had an uncatalyzed barrier of Δ*G*^‡^ = 25.8 kcal and did not require additional explicit water molecules (adding them increased Δ*G*^‡^). The hydride is transferred directly between the two carbons as the proton hops between the oxygens, as shown in Figure 3A. A catalyst molecule can be added in two possible positions: between the two carbons to facilitate the hydride transfer or between the carbonyl-O of CH_2_O and the hydroxyl of CH_2_(OH)_2_ to facilitate proton transfer. In all cases, the latter situation was favored.

Our calculated Δ*G*^‡^ values are shown in Figure 3B. Once again, we find that alcohols and H_2_S do not catalyze the reaction; the barriers are in the 30–34 kcal range (similar to adding one explicit water molecule where Δ*G*^‡^ is 29.5 kcal). The carboxylic acids and NH_3_ lower the barrier by 4–8 kcal, a similar range to what we found in formaldehyde hydration. When NH_3_ is a catalyst, the bond distances suggest that it abstracts a proton from CH_2_(OH)_2_ early in the process (the O…H distance is 1.39 Å while the forming N–H bond has already shortened to 1.14 Å). The hydride is being transferred (C…H distances of 1.31 and 1.40 Å), but no proton transfer is yet observed from NH_3_ to the carbonyl-O of CH_2_O. This mimics a base-catalyzed reaction. It also suggests that one way to get around the difficulty of explicitly including hydroxide (in an anionic transition state) is to utilize NH_3_ as a proxy.

The structure with HCOOH is interesting. The hydride is being transferred (C…H distances of 1.32 and 1.36 Å) in concert with the proton transfer between HCOOH and CH_2_O (O…H distances of 1.22 and 1.18 Å). The abstraction of the proton from CH_2_(OH)_2_ is only barely beginning (the O–H bond has only marginally lengthened to 1.05 Å). Is this an acid-catalyzed reaction? Probably not; it looks mostly like the water-only transition state, except that HCOOH facilitates the process and lowers Δ*G*^‡^. This illustrates why we should be cautious in interpreting our transition states and barriers. Molecular HCOOH acts as a proxy catalyst by lowering the barrier, but this does not imply an acid-catalyzed reaction. While Cannizzaro reactions are catalyzed by a strong base, we have not found any reference to acid catalysis, although we find one reference of the uncatalyzed reaction in aqueous solution proceeding at high temperature and high pressure [38].

In a buffered pH of ~7, NH_3_ exists primarily as ammonium, and HCOOH is in its carboxylate form in solution. Thus, we emphasize that in our calculations, molecular NH_3_ and HCOOH are acting as *proxy* catalysts. If there was a microenvironment in which the reactants and our neutral molecular “catalysts” are present, and they collide in the right orientation with sufficient energy, then the barrier might be lower in that instance. But in bulk quantities in an actual experiment at a particular pH, these specific molecules might not be catalytic. Thus, looking at transition state structures is important; only then can we see that HCOOH is acting as a proxy for an acid-catalyst in formaldehyde hydration but not in the Cannizzaro reaction. Conversely, NH_3_ is acting as a proxy for a base-catalyst in the Cannizzaro reaction but not in formaldehyde hydration. Both molecules lead to lowered Δ*G*^‡^ in both reactions. We will revisit this issue in our concluding remarks.

### 3.3. Glycolaldehyde Reactions: Enolization, Aldol, Hydration, and Cannizzaro Reactions

Glycolaldehyde is the linchpin molecule of the smallest autocatalytic cycle in the formose reaction. Access to larger sugars comes from the feasibility of aldol additions; thus, the simplest aldol addition involves glycolaldehyde reacting with formaldehyde to form the C_3_ sugar glyceraldehyde. We will only consider the formation of D-glyceraldehyde. Its mirror image would have the same free energy; thus, the thermodynamics do not change (and we expect equal amounts of D and L sugars to be formed in this scenario, as observed experimentally in the formose reaction if no chiral catalysts are added). Transition states for aldol addition to form the enantiomers are also mirror images, so the barriers should also be equal. The aldol addition is feasible because glycolaldehyde is enolizable. Thus, we first examine the enolization reaction.

**Figure 4:**
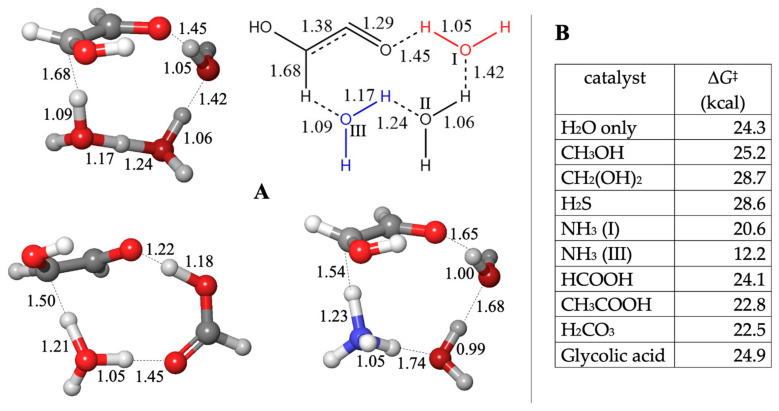
Transition state structures (**A**) and barriers (**B**) for the C_1_ + C_2_ → C_3_ aldol reaction.

The enolization reaction has Δ*G* = +6.8 kcal, and the uncatalyzed barrier is 24.3 kcal. As shown in Figure 4A, the optimal 10-center transition state utilizes three water molecules. The C–H bond breaks first (C…H is 1.68 Å), and the waters at positions II and III resemble a H_5_O_2_^+^ moiety. At the other end (involving water at position I), the O…H is just starting to form at 1.45 Å. Placing the alcohols and H_2_S in position I gives the lowest barriers, but just like before, these have higher Δ*G*^‡^, as shown in Figure 4B. The optimal position for carboxylic acids is to replace waters II and III. The C–H bond has not stretched as far (at 1.50 Å), while at the other end, the proton transfer is midway (O…H distances are 1.22 and 1.18 Å). The Δ*G*^‡^ values are similar to the water case. If NH_3_ replaced water in position I, the results are similar (with a marginally lower Δ*G*^‡^), but the barrier is significantly lowered (to 12.2 kcal) if NH_3_ is in position III. The C…H distance is 1.54 Å, and it looks like NH_3_ has mostly abstracted the hydrogen but not yet relayed it through the hydrogen bond network.

In the aldol addition, CH_2_O adds to the enol of glycolaldehyde, forming a C–C bond. The product formed is glyceraldehyde. Since the enol is a transient species, our Δ*G* and Δ*G*^‡^ values for the reaction are with reference to the aldehydes.

CH_2_O + C_2_H_4_O_2_ → C_3_H_6_O_3_ Δ*G* = −9.4 kcal

**Figure 5:**
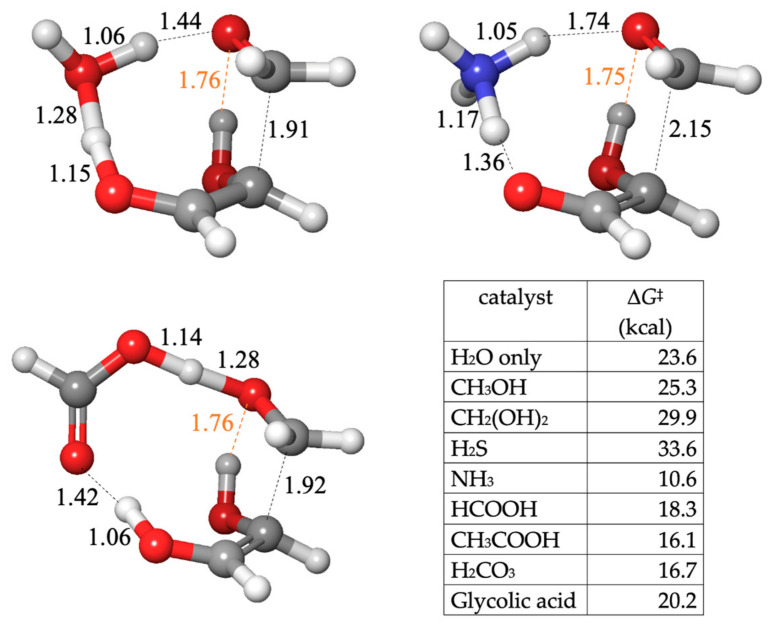
Transition state structures and barriers for the C_1_ + C_2_ → C_3_ aldol reaction.

The reaction is exergonic and remains so even if applied to the aldehyde-hydrates. Unlike the thermodynamic reversibility of oligomers forming and breaking C–O bonds, the equilibrium clearly favors C–C bond formation, as analyzed in our previous work [19]. As to the kinetics, our calculations yield Δ*G*^‡^ = 23.6 kcal for the uncatalyzed reaction. As shown in Figure 5, the forming C…C distance is 1.91 Å. The proton transfer distances are similar to what we observed in previous reactions. There is a hydrogen bond (dashed orange line) that does not directly participate in bond breaking/forming.

Similar to previous cases, the alcohols and H_2_S have either similar or higher barriers. The carboxylic acids have Δ*G*^‡^ values in the 16–20 kcal range. From Figure 5 (HCOOH), the C…C distance is similar, while the O…H distances favor proton transfer from the acid to CH_2_O over the enol-to-carbonyl proton transfer. The base-catalyzed features are more apparent with NH_3_ (with a very low barrier of 10.6 kcal): A proton is pulled early from the enol to form the enolate while the C…C forming bond is more distant at 2.15 Å. The NH_4_^+^ moiety has not begun to transfer a proton back to CH_2_O (the H…O distance is 1.74 Å). As we saw in the Cannizzaro reaction, NH_3_ acts as an effective proxy for base-catalyzed mechanisms.

At this point, we have considered four distinct reaction mechanisms: C–O bond formation (hydration), C–C bond formation (aldol), non-redox H-shifts via enolization, and redox H-shifts in the Cannizzaro disproportionation. Because the remaining reactions in this paper will recapitulate similar transition states, we will focus our attention mainly on NH_3_ and HCOOH as proxy catalysts and compare these to only having water molecules as a proton shuttle.

Glycolaldehyde hydration is similar to formaldehyde hydration, except the thermodynamics are less favorable. Our calculated Δ*G* for hydration is +0.5 kcal. This may seem surprising because one might expect hydration to be favored and for the reaction to be exergonic in a dilute solution. Indeed, if we include concentration corrections for a 1 M aqueous solution of glycolaldehyde, equilibrium slightly favors the hydrate. We have extensively analyzed the oligomers of glycolaldehyde in solution, and our computational protocol is in excellent agreement with NMR measurements [22], but in that paper, we also discuss why reporting concentration-corrected Δ*G* values can be misleading in a messy system where the concentrations of different reactants, intermediates, and products can range over many orders of magnitude. Thus, in this work (and in our previous free energy maps), we report our Δ*G* values without concentration corrections. Our uncorrected values provide a baseline for subsequent kinetic simulations that are beyond the scope of the present work. In our previous work, the barrier for glycolaldehyde hydration was 16.1 kcal, in good agreement with experimental results [22]. In the present work, HCOOH lowers the barrier to 11.0 kcal, while NH_3_ lowers it to 10.1 kcal. This is similar to what we found for CH_2_O hydration discussed earlier.

**Figure 6:**
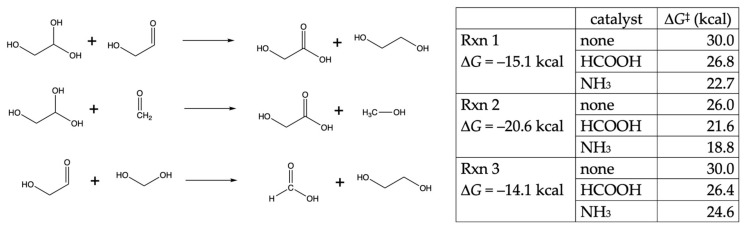
Cannizzaro reactions of glycolaldehyde.

Similar to formaldehyde, glycolaldehyde can also participate in Cannizzaro reactions. These may be self-disproportionation reactions (Rxn 1 in Figure 6), although more likely to be cross-Cannizzaro reactions between a C_1_ and C_2_ species (Rxn 2 and Rxn 3 in Figure 6) since formaldehyde (and its hydrate) is likely to be found in higher concentrations in the early stages of the formose reaction. The Δ*G* and Δ*G*^‡^ values of Rxn 2 are similar to the C_1_ Cannizzaro reaction discussed previously. The Δ*G* and Δ*G*^‡^ values of Rxn 1 and Rxn 3 are similar but ~4–6 kcal higher than Rxn 2.

These results are not surprising from our thermodynamic map of CHO compounds [20]. Essentially, the reaction exergonicity is driven by the relative stability of CH_2_O to CH_3_OH (18 kcal) versus the relative stability of glycolaldehyde to ethylene glycol (13 kcal). Since the transition states are similar, Hammond’s postulate suggests we will see similar differences in the barriers. Our results do imply that once the C_1_ food runs out, Cannizzaro reactions are less favored in the larger species; similar to the C_2_ case, the relative stability is ~14 kcal in both the C_3_ (glycolaldehyde to glycerol) and C_4_ cases (erythrose to erythritol). Thus, reduction of the C_1_ species (CH_2_O to CH_3_OH) is favored in the cross-Cannizzaro.

### 3.4. Glyceraldehyde Reactions: Isomerization and Aldol Addition

**Figure 7:**
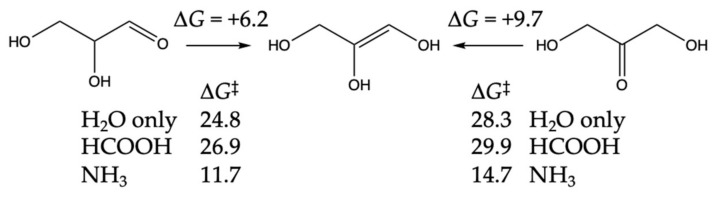
Enolization of glyceraldehyde and dihydroxyacetone. Δ*G* and Δ*G*^‡^ values in kcal.

Glyceraldehyde favorably isomerizes into dihydroxyacetone in aqueous solution. Our calculated Δ*G* for the reaction is −3.5 kcal, and the uncatalyzed barrier is 24.8 kcal (very similar to the 24.3 kcal enolization barrier for glycolaldehyde). The isomerization is a two-step reaction going through an enol intermediate. Figure 7 shows the Δ*G* and Δ*G*^‡^ values for enol formation. Since dihydroxyacetone is more stable than glyceraldehyde, these values are 3.0–3.5 kcal higher starting from dihydroxyacetone. The enolization barriers of glyceraldehyde with HCOOH and NH_3_ are very similar to those for glycolaldehyde, as expected, since the transition states are structurally similar. Uncatalyzed, the isomerization is relatively slow, but lower barriers with the proxy catalyst NH_3_ allow for more rapid interconversion. Dihydroxyacetone could be one way to sequester glyceraldehyde.

**Figure 8:**
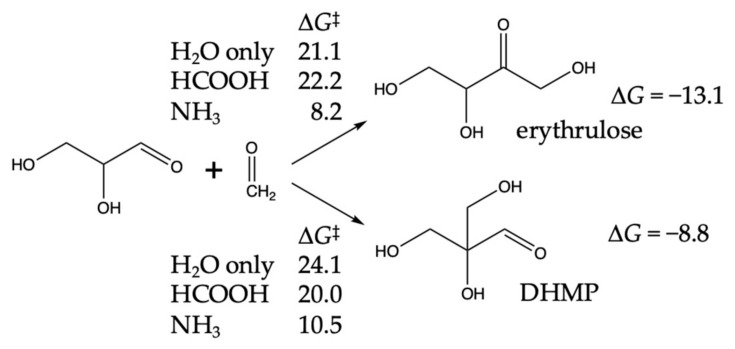
C_3_ + C_1_ → C_4_ aldol addition. Δ*G* and Δ*G*^‡^ values in kcal.

For the C_3_ + C_1_ → C_4_ aldol addition, we had previously determined [19] that the path to forming the ketone erythrulose had lower (uncatalyzed) barriers compared to forming the aldehydes, erythrose, or threose. However, we had not considered the formation of a branched tetrose 2,3-dihydroxy-2-(hydroxymethyl)propanal, which we will refer to as DHMP. Our results are shown in Figure 8.

For erythrulose formation, Δ*G* for the reaction is −13.1 kcal. While this is 4 kcal more exergonic than C_2_ + C_1_ → glyceraldehyde, it is because erythrulose is more stable than its aldehyde counterparts erythrose and threose by 4.2 and 3.5 kcal, respectively. The water-only barrier is 21.1 kcal, or 3.3 kcal lower than C_2_ + C_1_ → glyceraldehyde. While we expected the barrier with the HCOOH proxy catalyst to be lower than 21 kcal, this was not the case even after trying multiple conformations. (We did find a lower barrier with CH_3_COOH.) The barrier with the NH_3_ proxy catalyst is lowered by 13 kcal, a similar magnitude compared to C_2_ + C_1_ → glyceraldehyde.

DHMP formation is less exergonic. Steric hindrance destabilizes DHMP by ~1 kcal compared to its unbranched counterparts, erythrose and threose. The barriers for water-only, HCOOH, and NH_3_ closely matched what we found for C_2_ + C_1_ → glyceraldehyde.

Since glyceraldehyde can either convert into dihydroxyacetone or undergo aldol addition with CH_2_O to form erythrulose, and in both cases, our transition states go through the C_3_ enediol intermediate, there is competition between the two reactions. The aldol addition is thermodynamically favored with Δ*G* = −13.1 kcal compared to the isomerization with Δ*G* = −3.5 kcal (uphill 6.2 to the enediol, then downhill 9.7 to the ketone). The aldol reaction is also kinetically favored. From the enediol, the water-only aldol addition has Δ*G*^‡^ = 21.1 − 6.2 = 14.9 kcal, while the enol-to-keto isomerization has Δ*G*^‡^ = 28.3 − 9.7 = 18.6 kcal. Thus, we would expect aldol addition to outcompete enol-to-keto isomerization when CH_2_O is plentiful. (NH_3_ lowers the barriers for both reactions by similar amounts; thus, aldol addition is still favored.) This fits with what Appayee and Breslow found in their follow-up experiments [3] prompted by work in Benner’s group [39]; at higher concentrations of CH_2_O, hardly any dihydroxyacetone is observed, but at lower concentrations, enolization can come into play.

**Figure 9:**
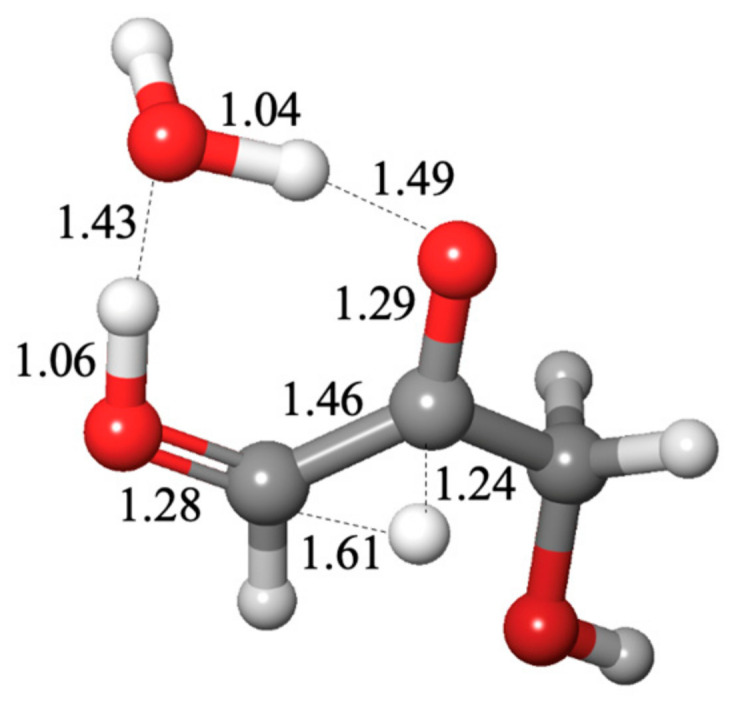
Transition state of 1,2-hydride shift for glyceraldehyde-dihydroxyacetone isomerization.

Additionally, Appayee and Breslow [3] proposed a 1,2-hydride shift for the isomerization of glyceraldehyde to dihydroxyacetone based on deuterium labeling experiments. According to their mechanism, this conversion is catalyzed by Ca^2+^ and hydroxide ions. Because our protocol is limited to neutral species (as discussed in Section 2), we can only investigate this with neutral molecule catalysts. The optimal transition state has one mediating water molecule between the oxygens, as shown in Figure 9. The proton from the 2-hydroxy group has been mostly transferred (H…O is 1.49 Å), while the middle C…H (at 1.24 Å) is only just starting to break. This is somewhat analogous to Appayee and Breslow’s proposed mechanism, where deprotonation takes place before the deuterium transfer. However, our calculated Δ*G*^‡^ is 30.0 kcal, which is higher than enolization. Adding HCOOH or NH_3_ in place of the mediating water molecule does not lower the calculated barrier.

Because our calculations do not mimic the most productive experimental formose reaction conditions for sugar synthesis (high pH, the presence of divalent metal ions or borate, higher temperatures, and reactant concentrations), they limit the mechanisms we can explore, and we cannot always make direct comparisons to experimental results. However, our consistent protocol allows us to peg the energies of the myriad compounds that may show up in a messy prebiotic mixture. This approach has allowed us to generate a series of intersecting free energy maps under a set of baseline conditions for a wide range of prebiotic systems. And while our protocol has its limitations, we have some confidence that our calculated free energies are reasonable and can be validated, as discussed in Section 2.

### 3.5. Tetrose Reactions: Isomerization, Ring Closure, and Retro-Aldol Splitting

While erythrulose is the most stable of the tetrose sugars, it does not easily undergo retro-aldol splitting to form glycolaldehyde. The key step of re-forming the C_2_ linchpin facilitates autocatalysis in the formose reaction. Instead, it is the aldehyde that more favorably undergoes the retro-aldol reaction; thus, erythrulose has to first isomerize into erythrose or threose. For the enolization of erythrulose, we calculate Δ*G* to be +9.7 kcal, essentially matching the enolization of its C_3_ counterpart, dihydroxyacetone. The barriers are also similar: Δ*G*^‡^ values are 29.4, 28.7, and 13.0 kcal for the water-only, HCOOH, and NH_3_ proxy catalysts. (See Figure 7 for the counterpart C_3_ values).

The linear C_4_ aldehydes can potentially be sequestered by ring closure reactions and thus less susceptible to retro-aldol reactions. We previously calculated the free energy changes for both D-erythrose and D-threose into their α- and β-furanoses [19]; the linear tetroses and their furanoses are 1 kcal or less apart, so we expect both to be present at equilibrium. Since ring closure and opening resemble hydration and dehydration, it is not surprising that the barriers are similar in magnitude.

**Figure 10:**
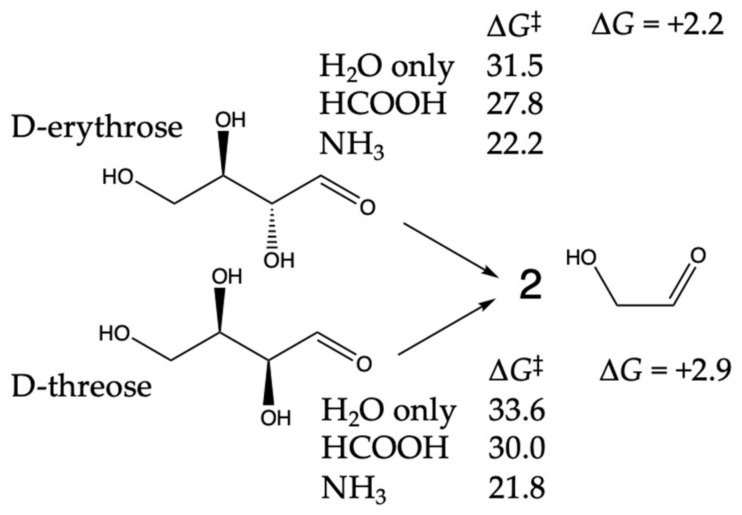
C_4_ → C_2_ + C_2_ retro-aldol splitting. Δ*G* and Δ*G*^‡^ values in kcal.

We had not considered the retro-aldol C_4_ → C_2_ + C_2_ in our previous work, so we will examine it more carefully here. Our results are shown in Figure 10. The retro-aldol splitting is marginally endergonic. Since D-threose is more stable than D-erythrose by 0.7 kcal, the retro-aldol of D-threose is 0.7 kcal more endergonic, and its corresponding barriers are also marginally higher. At over 30 kcal/mol for the water-only barriers, the uncatalyzed retro-aldol is expected to be quite slow at room temperature. HCOOH and NH_3_ proxy catalysts lower Δ*G*^‡^, but these values are still over 20 kcal/mol. It is not surprising that the retro-aldol (being endergonic) has higher barriers than the (exergonic) aldol additions.

**Figure 11:**
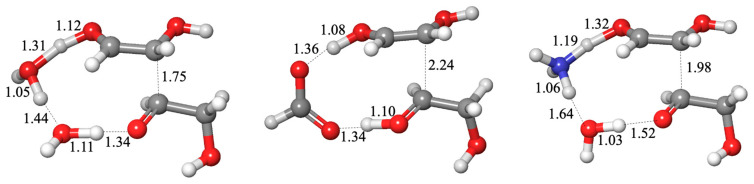
Transition state structures for retro-aldol splitting.

The three retro-aldol transition states for D-erythrose are shown in Figure 11. For water-only, the breaking C–C bond at 1.75 Å has not extended very far, while the O…H distances suggest that the proton hops between water and erythrose have proceeded substantially. For HCOOH, the acid proton has mostly hopped to the aldehyde carbonyl (O–H is 1.08 Å), and the breaking C–C bond is substantially longer at 2.24 Å. For NH_3_, the aldehyde carbonyl is starting to grab a proton (O…H is 1.32 Å), and that is because NH_3_ has already grabbed a proton (N–H is 1.06 Å) from the neighboring water, which has also grabbed its proton (O–H is 1.03 Å) from the alcohol group on erythrose.

### 3.6. Sequestering and Parasitic Reactions

Earlier, we discussed how CH_3_OH, produced in the C_1_ Cannizzaro reaction, could sequester CH_2_O via acetal formation. In this section, we examine possible reactions that may either sequester or remove “food” or intermediates from the smallest autocatalytic cycle. Most of our analysis will focus on the C_1_ compounds, and we will discuss extrapolations to the C_2_, C_3,_ and C_4_ sugars in the cycle.

We saw earlier that the addition of water to CH_2_O to form the hydrate CH_2_(OH)_2_ was exergonic 4.6 kcal with a barrier of 13.2 kcal. What if instead of acting as proxy catalysts, NH_3_ or H_2_S could add to CH_2_O in a similar way? (The transition states both include two additional water molecules facilitating proton transfer.)

CH_2_O + NH_3_ → CH_2_(OH)(NH_2_) Δ*G* = −4.6 kcal Δ*G*^‡^ = 3.9 kcal

CH_2_O + H_2_S → CH_2_(OH)(SH) Δ*G* = −3.9 kcal Δ*G*^‡^ = 12.1 kcal

Both reactions are exergonic. CH_2_(OH)(NH_2_) is similar in relative energy to the hydrate, while CH_2_(OH)(SH) is higher by 0.7 kcal. Since the barrier for NH_3_ addition is significantly lower in the presence of NH_3_, we expect NH_3_ to favorably react with aldehydes if present in the solution. H_2_S is marginally disfavored and, unless present in high concentration, is less likely to be found added to CH_2_O. The dominant species in aqueous solution is still CH_2_(OH)_2_.

Since CH_3_OH may be generated from Cannizzaro reactions, NH_3_ and H_2_S could potentially act as attacking nucleophiles in an S_N_2 reaction.

CH_3_OH + NH_3_ → CH_3_NH_2_ + H_2_O Δ*G* = −2.8 kcal Δ*G*^‡^ = 32.2 kcal

CH_3_OH + H_2_S → CH_3_SH + H_2_O Δ*G* = −8.1 kcal Δ*G*^‡^ = 20.6 kcal

Both reactions are exergonic, but the barriers are higher. Recall from Section 2 that our S_N_2 transition states require a daisy chain of 3–4 additional water molecules, and in our experience, our calculated barriers are artificially high. (We calculate Δ*G*^‡^ = 43.3 kcal for the self-barrier for CH_3_OH + H_2_O.) In a dilute aqueous solution, the equilibrium may shift toward hydrolyzing CH_3_NH_2_, but the exergonicity of CH_3_SH formation suggests that it might persist in solution as a potential reactant. The lower Δ*G*^‡^ for H_2_S addition suggests that CH_3_SH formation might be quite facile in a messy formose system spiked with H_2_S.

Since HCOOH may also be present from Cannizzaro reactions, nucleophilic addition of NH_3_, H_2_S, and CH_3_OH (with subsequent elimination of water as a leaving group) could respectively lead to amides, thioacids, and esters. (Since this is a two-step reaction, we report Δ*G*^‡^ for the rate-determining step.)

HCOOH + NH_3_ → HC(O)NH_2_ + H_2_O Δ*G* = –5.4 kcal Δ*G*^‡^ = 24.1 kcal

HCOOH + H_2_S → HC(O)SH + H_2_O Δ*G* = +10.0 kcal Δ*G*^‡^ = 33.1 kcal

HCOOH + CH_3_OH → HCO_2_CH_3_ + H_2_O Δ*G* = –0.4 kcal Δ*G*^‡^ = 26.3 kcal

Amide formation is exergonic, as expected, and the barrier is moderate. Once amides are formed, their hydrolysis (with a barrier of ~30 kcal) is slower. Formamide, the C_1_ product of this reaction, is prominent in prebiotic chemistry scenarios [40,41]. We have previously computed the route to formamide via the hydrolysis of HCN, which is more exergonic but has a higher barrier [42]. On the other hand, forming the thioacid is rather endergonic, and this contributes to its higher barrier. From our recent work mapping the free energies of small CHOS compounds [43], we find that thioester formation is less endergonic (6–7 kcal), and thus, coupled with the stability of CH_3_SH as a reactant, a feasible route to thioesters may be envisioned. With regard to ester formation, our calculated Δ*G* close to zero suggests that in a relatively dilute aqueous solution, the equilibrium favors hydrolysis.

While C_1_ food is plentiful, the possible presence of CH_2_(OH)(NH_2_) and CH_2_(OH)(SH) in solution opens up the possibility of additional Cannizzaro reactions from the favorable reduction in CH_2_O to CH_3_OH.

CH_2_O + CH_2_(OH)(NH_2_) → HC(O)NH_2_ + CH_3_OH Δ*G* = –25.0 kcal Δ*G*^‡^ = 23.3 kcal

CH_2_O + CH_2_(OH)(SH) → HC(O)SH + CH_3_OH Δ*G* = –10.2 kcal Δ*G*^‡^ = 28.9 kcal

In this third route to formamide, the reaction exergonicity doubly benefits from the stability of the amide and the reduction to CH_3_OH. The barrier is similar to the self-disproportionation of formaldehyde and its hydrate, which was reported earlier. Unlike the aforementioned route to the thioacid, the Cannizzaro reaction, in this case, is exergonic, although it also has a high barrier. (This suggests a promising route for thioester formation, and we are generating several energy maps utilizing CH_3_SH as a reactant and expect to present those results in our next publication.)

Looking at this small subset of reactions suggests that the possibilities multiply exponentially as we go beyond C_1_ compounds. For the C_2_ compounds, besides glycolaldehyde and its hydrate, we might see glycolic acid and ethylene glycol as first-order Cannizzaro products, but subsequent redox reactions might lead to acetic acid and ethanol. All these compounds can react with each other (and with NH_3_, H_2_S, CH_3_NH_2_, CH_3_SH, etc.), leading to a plethora of compounds—a very messy system! We expect to pursue some of the promising reactions in the near future, but since we have been examining the smallest autocatalytic cycle, we close this section by presenting our results on the thermodynamics for adding water, NH_3_, and H_2_S to the carbonyl group of the C_2_, C_3,_ and C_4_ sugars.

The results in Table 1 suggest that reaction with NH_3_ can potentially sequester the aldehydes, but the reaction is reversible, and the barriers are likely to be very low (as we saw for NH_3_ adding to CH_2_O). H_2_S is less effective as a sequestering agent. For the ketones, the addition reactions are all endergonic, and we do not expect sequestration to be a factor. If the aldehyde-NH_3_ adducts subsequently participate in exergonic Cannizzaro reactions, these are unlikely to be reversible and, therefore, parasitic on the cycle. While the barriers are expected to be significant (23 kcal in the case of C_1_), they are not insurmountable at moderate to higher temperatures.

## 4. Overall Free Energy Map and Concluding Remarks

**Figure 12:**
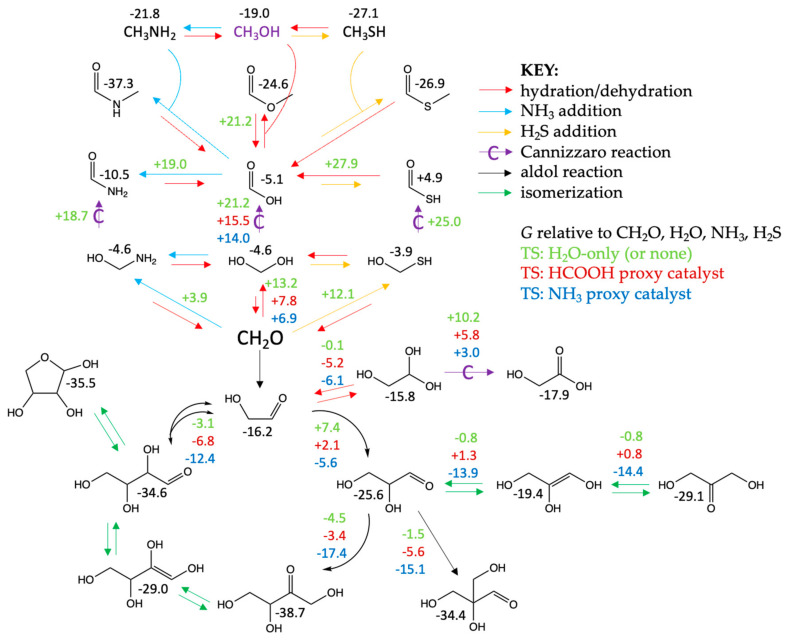
Overall free energy map with *G* values in kcal relative to CH_2_O, H_2_O, NH_3_, and H_2_S.

In the previous section, we discussed the reactions of C_1_, C_2_, C_3,_ and C_4_ “sugars” piecemeal with Δ*G* and Δ*G*^‡^ values for each individual reaction. In this section, we will look at the overall free energy map. With multiple pathways, we have found it easier to present our results, not along a particular reaction coordinate but with a bird’s-eye-view schematic, as shown in Figure 12. For this to be effective, we need to choose a set of reference compounds and assign them relative free energies of zero. Our reference compounds will be CH_2_O, H_2_O, NH_3,_ and H_2_S.

The numbers in black and colored fonts are for stable minima and transition states, respectively. The *G* value for CH_2_O in the center of Figure 12 is zero. At a glance, you can see the relative stability of each compound with respect to the reference states. Making new C–C bonds via aldol additions is significantly exergonic. For example, the reaction of glycolaldehyde (*G* = −16.2) with formaldehyde to form glyceraldehyde (*G* = −25.6 kcal) has a Δ*G* of −25.6 − (−16.2 + 0) = −9.4 kcal as shown in Section 3.3. The uncatalyzed (H_2_O-only) transition state (*G* = +7.4 in green font) tells us that the forward barrier Δ*G*^‡^ is +7.4 − (−16.2) = +23.8 kcal while the reverse barrier would be Δ*G*^‡^ = +7.4 − (−25.6) = +33.2 kcal. For the HCOOH catalyzed reaction (transition state *G* = +2.1 in dark red font), the forward barrier Δ*G*^‡^ is +2.1 − (−16.2) = 18.3 kcal (as shown in Figure 5) while the reverse barrier would be Δ*G*^‡^ = +2.1 − (−25.6) = +27.7 kcal.

As a second example, consider the reaction of HCOOH (*G* = −5.1) reacting with H_2_S (*G* = 0, a reference compound) to form the thioacid HC(O)SH (*G* = +4.9). Δ*G* of this reaction is +4.9 − (−5.1 + 0) = +10.0 kcal. On the other hand, the reaction of HCOOH with CH_3_SH (*G* = −27.1) to form the thioester HC(O)SCH_3_ (*G* = −26.9) has Δ*G* = −26.9 − (−5.1 −27.1) = +5.3 kcal. Hence, forming the thioester is less endergonic than forming the thioacid, as discussed in Section 3.6. Except for the exergonic formation of amides, the sequestering reactions (top half of Figure 12) have Δ*G* close to zero or slightly endergonic.

Figure 12 is only a very small part of what may take place in a messy formose reaction if NH_3_ and H_2_S are present. All the reactions in the top half only show reacting C_1_ species, but because the formose reaction generates C_2_, C_3,_ and C_4_ aldehydes, ketones, alcohols, and carboxylic acids (the latter two via Cannizzaro reactions), these can further react with each other and with NH_3_ and H_2_S. While we do not have all these calculations completed, what we have presented thus far allows us to make reasonable first approximations of both the thermodynamics and kinetics of these myriad reactions. We expect to set up kinetic simulations to explore potential product distributions in this complex system in a future publication when we have accumulated more data.

Figure 12 also summarizes most of the results we have discussed in the previous section, and we hope this has provided the reader with a free energy “map” to see where the hills and valleys are located, how high the hills are, and how deep or shallow the valleys lie. The takeaway summary: (1) C–C bond-forming aldol reactions are thermodynamically more favorable than C–O, C–S, or C–N bond formation. (2) Kinetically, C–N bond formation has the lowest barriers. (3) Cannizzaro reactions open access to species with other redox states and are thermodynamically driven by forming the reduced (alcohol) product, although the kinetic barriers are relatively high. (4) HCOOH and NH_3_ can be used as proxy catalysts in computational studies; looking at the transition states gives us a sense of whether a reaction may be acid- or base-catalyzed, but we should be cautious not to over-interpret the results. (5) The exponentially growing set of compounds in the formose reaction includes many opportunities for some intermediates to act as catalysts, sequestering agents, or even parasites of the autocatalytic cycle. Our study only touches a very small part of the initial steps that may occur, but we hope it serves as a guide for future studies.

A big origins-of-life question is how and if an autocatalytic cycle of (sugar) aldehydes evolved into a proto-metabolic cycle of carboxylic acids analogous to the reverse TCA cycle. Kinetically, the main impact of HCOOH is catalyzing the disproportionation of aldehydes to form more acids, parasitizing the formose cycle, while furnishing acids via Cannizzaro reactions toward constructing an analogous cycle of carboxylic acid proto-metabolites. HCOOH has little impact on the enolization and aldol additions required to form larger compounds. On the other hand, NH_3_ lowers the barriers of all reactions and acts as an indiscriminate catalyst. By doing so, it allows thermodynamics to play a role in favoring the formation of larger molecules (potential biomass) via new C–C bonds in aldol additions. Thermodynamically, amide bonds are also more stable. This may hint at some roles of nitrogen in extant life today; we find them in biocatalytic proteins or in secondary metabolites, but they are not metabolites in the core pathways, such as glycolysis and the TCA cycle. The eventual evolution to a carboxylic acid cycle with higher barriers now requires catalysts to proceed (because aldol reactions are no longer as feasible kinetically), thus leading to selectivity and control in metabolism.

## Figures and Tables

**Table 1:** Thermodynamics of addition reactions to sugar carbonyls.

Sugar	Added Molecule	Δ*G* (kcal)
Glycolaldehyde	H_2_O	+0.4
	NH_3_	−0.4
	H_2_S	+2.6
Glyceraldehyde	H_2_O	−0.1
	NH_3_	0.0
	H_2_S	+2.2
Dihydroxyacetone	H_2_O	+2.6
	NH_3_	+3.7
	H_2_S	+7.2
Erythrose	H_2_O	−0.4
	NH_3_	−0.7
	H_2_S	+2.8
Threose	H_2_O	+1.1
	NH_3_	+0.1
	H_2_S	+3.5
Erythrulose	H_2_O	+3.8
	NH_3_	+2.8
	H_2_S	+6.0

## Data Availability

The data presented in this study are available in Supplementary Materials.

## Supplementary Materials

The following supporting information can be downloaded at https://www.mdpi.com/article/10.3390/life14080933/s1, Table S1: Energy breakdown of molecules and transition states.
