# Supplementary material for: Exploring the Core Formose Cycle: Catalysis and Competition
Source: Life (Basel). 2024 Jul 25;14(8):933. doi: 10.3390/life14080933 (PMC11355428; doi:10.3390/life14080933)
Supplement: Supplementary file 1 [file life-14-00933-s001.zip › life-3083248-supplementary.pdf]

Supporting Information for:

Exploring the Core Formose Cycle: Catalysis and Competition

By Jeremy Kua* & L. Philip Tripoli
University of San Diego, 5998 Alcala Park, San Diego, CA 92110, U.S.A.
* jkua@sandiego.edu

Table S1 contains the energy breakdown for calculated structures in this article. The computational methods section describes each of these categories: E_{elec} is the electronic energy in atomic units. All other values are in kcal/mol. E_{solv} is the solvation energy when the molecule is embedded in a dielectric. H_{corr} includes the zero-point-energy and standard enthalpy corrections to 298 K. The entropic correction ($-0.5TS_{corr}$) is half of the standard gas-phase entropy correction at 298 K. The last column (G_{298}) is the total free energy obtained by adding the other four columns (where the atomic units of E_{elec} are converted to kcal/mol by multiplying 627.5096. The transition states are labeled "TS".

Table S1. Energy breakdown of molecules and transition states

	E_{elec} (a.u.)	E_{solv}	H_{corr}	$-0.5TS_{corr}$	G_{298}
**Baseline (reference) molecules**					
H_2O	−76.44744	−8.66	15.74	−6.73	−47971.15
NH_3	−56.57604	−6.27	23.91	−6.86	−35491.23
H_2S	−399.42206	−1.29	11.76	−7.33	−250638.04
CH_2O	−114.53629	−2.75	19.01	−7.79	−71864.15
**Other catalyst molecules**					
CH_3OH	−115.75737	−5.50	34.74	−8.47	−72618.09
HCOOH	−189.81947	−6.96	23.81	−8.84	−119105.53
H_2CO_3	−265.08295	−11.91	27.70	−9.61	−166335.92
CH_3COOH	−229.14542	−11.19	41.38	−9.69	−143770.45
glycolic acid	−304.38296	−13.77	46.13	−10.86	−190981.73
**Formaldehyde hydration**					
$CH_2(OH)_2$	−191.00777	−10.30	38.94	−9.28	−119839.85
TS: H_2O only	−343.90414	−16.56	67.39	−12.01	−215764.33
TS: CH_3OH	−383.21333	−13.46	86.07	−13.46	−240410.89
TS: $CH_2(OH)_2$	−458.45901	−18.50	89.87	−14.07	−287630.13
TS: H_2S	−666.86922	−19.07	66.81	−13.00	−418432.10
TS: NH_3	−324.02722	−18.47	75.78	−12.45	−203285.33
TS: HCOOH	−380.81906	−12.97	59.54	−12.00	−238933.05
TS: H_2CO_3	−456.08745	−16.70	63.59	−12.72	−286165.08
TS: CH_3COOH	−496.62200	−16.98	94.73	−14.94	−311572.26
TS: glycolic acid	−571.84817	−24.51	98.69	−15.69	−358781.73
**C_1 Cannizzaro reaction**					
TS: no catalyst	−305.51325	−9.52	55.01	−11.15	−191678.16
TS: CH_3OH	−421.27923	−10.71	89.75	−14.05	−264291.77
TS: $CH_2(OH)_2$	−496.52623	−14.14	93.81	−14.52	−311509.83

TS: H_2S	−704.92963	−15.35	69.01	−13.22	−442309.67
TS: NH_3	−362.09686	−19.36	80.23	−13.15	−227171.54
TS: HCOOH	−495.35611	−14.22	79.49	−13.99	−310789.43
TS: H_2CO_3	−570.62450	−17.93	83.58	−14.65	−358021.35
TS: CH_3COOH	−534.69180	−13.70	97.58	−15.17	−335455.53
TS: glycolic acid	−609.91718	−20.10	101.46	−15.95	−382663.48
**Glycolaldehyde Enolization**					
glycolaldehyde	−229.11010	−7.23	41.58	−10.07	−143744.51
glycolaldehyde-enol	−229.09642	−9.16	41.54	−9.88	−143737.70
TS: H_2O only	−458.45370	−23.56	88.45	−14.43	−287633.64
TS: CH_3OH	−497.76287	−19.98	107.10	−15.86	−312279.72
TS: $CH_2(OH)_2$	−573.00994	−23.55	110.74	−15.85	−359497.90
TS: H_2S	−781.41167	−23.39	85.42	−15.21	−490296.50
TS: NH_3	−438.59540	−21.78	99.38	−15.14	−275160.36
TS: HCOOH	−495.36457	−15.38	78.49	−14.15	−310797.06
TS: H_2CO_3	−570.63236	−19.49	82.49	−14.79	−358029.07
TS: CH_3COOH	−534.70026	−15.39	96.93	−15.34	−335463.35
TS: glycolic acid	−609.92607	−22.60	100.65	−16.12	−382672.53
**Aldol Addition: $C_1 + C_2 \rightarrow C_3$**					
glyceraldehyde	−343.67597	−10.51	64.18	−11.78	−215618.08
TS: H_2O only	−496.55480	−15.64	94.68	−14.24	−311528.10
TS: CH_3OH	−459.39763	−7.37	95.97	−14.10	−288201.92
TS: $CH_2(OH)_2$	−534.64481	−10.43	100.54	−14.49	−335419.13
TS: H_2S	−743.03967	−9.44	73.55	−13.42	−466213.84
TS: NH_3	−400.22211	−14.55	86.36	−12.96	−251084.37
TS: HCOOH	−533.46627	−11.72	84.72	−14.14	−334696.35
TS: H_2CO_3	−608.73510	−15.20	88.74	−14.77	−381928.35
TS: CH_3COOH	−572.80265	−11.89	102.36	−14.78	−359363.47
TS: glycolic acid	−648.02776	−17.71	106.81	−16.08	−406570.62
**Glycolaldehyde Hydration**					
glycolaldehyde hydrate	−305.57860	−11.89	61.26	−10.98	−191715.12
TS: H_2O only	−458.47233	−18.97	89.33	−13.69	−287639.12
TS: HCOOH	−571.85414	−20.63	98.8	−15.52	−358781.31
TS: NH_3	−438.60074	−21.35	99.19	−14.12	−275162.45
**Glycolaldehyde Cannizzaro**					
ethylene glycol	−230.32320	−10.09	57.43	−10.32	−144493.00
Rxn 1 TS: no catalyst	−534.65732	−13.16	100.19	−14.03	−335429.60
Rxn 1 TS: HCOOH	−724.50226	−14.41	124.9	−16.73	−454538.36
Rxn 1 TS: NH_3	−591.24283	−21.59	125.19	−15.16	−370922.11
Rxn 2 TS: no catalyst	−420.08106	−12.57	77.15	12.95	−263527.37
Rxn 2 TS: HCOOH	−609.92930	−13.94	102.80	−15.59	−382663.22
Rxn 2 TS: NH_3	−476.66230	−24.08	102.79	−14.77	−299046.23
Rxn 3 TS: no catalyst	−420.07782	−15.35	77.40	−12.97	−263553.78
Rxn 3 TS: HCOOH	−609.93040	−14.17	103.69	−15.84	−382663.50
Rxn 3 TS: NH_3	−476.65653	−26.67	102.77	−15.06	−299045.51
**Glyceraldehyde Isomerization**					

glyceraldehyde enol	−343.67597	−10.51	64.18	−11.78	−215618.08
GLA to enol TS: H_2O only	−573.02682	−24.11	113.28	−16.01	−359506.67
GLA to enol TS: HCOOH	−609.92934	−17.35	102.03	−16.02	−382667.86
GLA to enol TS: NH_3	−553.15493	−28.89	121.55	−17.10	−347034.47
dihydroxyacetone	−343.67964	−11.22	63.96	−12.12	−215621.65
DHA to enol TS: H_2O only	−573.02264	−24.95	111.33	−15.99	−359506.82
DHA to enol TS: HCOOH	−609.93566	−14.67	102.16	−15.45	−382668.44
DHA to enol TS: NH_3	−553.16609	−23.75	122.35	−16.55	−347034.98
**Aldol Addition: $C_1 + C_3 \rightarrow C_4$**					
erythrulose	−458.24285	−16.42	86.48	−13.67	−287495.40
to erythrulose TS: H_2O only	−534.65634	−15.88	99.54	−14.45	−335432.78
to erythrulose TS: HCOOH	−648.03447	−14.48	107.10	−15.88	−406571.11
to erythrulose TS: NH_3	−514.78600	−18.54	108.51	−14.78	−322957.97
DHMP	−458.24006	−14.01	86.50	−13.54	−287491.09
to DHMP TS: H_2O only	−534.65228	−15.85	99.88	−14.36	−335429.77
to DHMP TS: HCOOH	−648.03076	−14.56	107.56	−15.76	−406568.28
to DHMP TS: NH_3	−514.79120	−18.09	108.89	−14.73	−322960.35
**Erythrulose Enolization**					
erythrulose enol	−458.22734	−16.88	86.87	−13.68	−287485.74
TS: H_2O only	−687.59372	−25.35	134.94	−17.36	−431379.43
TS: HCOOH	−724.49973	−20.32	124.55	−17.12	−454543.43
TS: NH_3	−667.72796	−29.40	142.66	−18.00	−418910.45
**Retro-aldol: $C_4 \rightarrow C_2 + C_2$**					
D-erythrose	−458.23911	−14.66	86.46	−13.58	−287491.22
from erythrose TS: H_2O only	−611.10861	−25.37	116.15	−16.31	−383402.05
from erythrose TS: HCOOH	−648.02183	−20.85	107.49	−16.38	−406569.66
from erythrose TS: NH_3	−591.23918	−27.30	125.76	−16.89	−370926.69
D-threose	−458.24058	−14.69	86.57	−13.44	−287491.92
from threose TS: H_2O only	−611.11664	−19.65	116.47	−15.90	−383400.64
from threose TS: HCOOH	−648.02047	−19.29	107.33	−16.36	−406567.39
from threose TS: NH_3	−591.24860	−22.07	125.90	−16.66	−370927.00
**Sequestering/Parasitic Reactions**					
$CH_2(OH)(NH_2)$	−171.13226	−9.99	46.68	−9.51	−107359.96
TS: H_2O only	−324.03657	−16.66	74.47	−12.46	−213964.96
$CH_2(OH)(SH)$	−513.97545	−7.11	35.57	−10.01	−322506.07
TS: H_2O only	−666.86943	−16.85	64.59	−13.11	−418432.34
CH_3NH_2	−95.88842	−4.24	42.77	−8.56	−60140.93
TS: H_2O only (S_N2)	−401.67275	−31.38	110.03	−15.68	−251998.67
CH_3SH	−438.74268	−0.42	31.66	−9.03	−275393.03
TS: H_2O only (S_N2)	−744.52594	−34.97	99.50	−16.32	−467148.96
$HC(O)NH_2$	−169.94622	−10.33	31.38	−9.18	−106631.01
TS: H_2O only (rate-determining)	−399.29394	−19.49	78.47	−13.16	−250514.96
HC(O)SH	−512.77342	−2.97	20.30	−9.43	−321762.34
TS: H_2O only (rate-determining)	−742.12620	−16.36	68.44	−13.48	−465652.71
HC(O)OH	−189.81947	−6.96	23.81	−8.84	−119105.53
TS: H_2O only (rate-determining)	−458.47472	−16.61	89.01	−14.11	−287638.99

**Adducts from Table 1**					
glycolaldehyde•H_2O	−305.57553	−13.62	61.05	−11.12	−191715.27
glycolaldehyde•NH_3	−285.69998	−14.39	68.93	−11.26	−179236.20
glycolaldehyde•H_2S	−628.53939	−11.65	57.83	−11.69	−394380.01
glyceraldehyde•H_2O	−420.14287	−16.33	83.53	−12.80	−263589.28
glyceraldehyde•NH_3	−400.26677	−17.00	91.56	−12.72	−251109.52
glyceraldehyde•H_2S	−743.10726	−14.06	80.39	−13.29	−466253.90
dihydroxyacetone•H_2O	−420.14643	−15.07	83.28	−12.66	−263590.36
dihydroxyacetone•NH_3	−400.26790	−15.78	91.21	−12.82	−251109.33
dihydroxyacetone•H_2S	−743.10646	−12.72	80.01	−13.32	−466252.46
erythrose•H_2O	−534.70580	−21.14	105.83	−14.46	−335462.79
erythrose•NH_3	−514.83994	−16.48	114.31	−13.90	−322983.07
erythrose•H_2S	−857.67192	−16.71	102.59	−14.96	−538126.44
threose•H_2O	−534.70837	−19.22	106.04	−14.21	−335462.03
threose•NH_3	−514.83470	−19.01	113.86	−14.18	−322983.05
threose•H_2S	−857.67231	−16.94	102.81	−14.79	−538126.52
erythrulose•H_2O	−534.70832	−19.46	105.63	−14.18	−335462.61
erythrulose•NH_3	−514.84129	−15.95	113.95	−13.83	−322983.68
erythrulose•H_2S	−857.67186	−15.23	102.46	−14.81	−538124.91
